# Disordered region of H3K9 methyltransferase Clr4 binds the nucleosome and contributes to its activity

**DOI:** 10.1093/nar/gkz480

**Published:** 2019-06-05

**Authors:** Elias Akoury, Guoli Ma, Segolene Demolin, Cornelia Brönner, Manuel Zocco, Alexandre Cirilo, Nives Ivic, Mario Halic

**Affiliations:** 1 Department of Biochemistry, Gene Center, Ludwig-Maximilians-Universität LMU, Feodor-Lynen-Strasse 25, 81377 Munich, Germany; 2 Department of Chemistry, Faculty of Chemistry and Pharmacy, Ludwig-Maximilians-Universität LMU, Butenandtstrasse 5-13, 81377 Munich, Germany; 3 Department of Natural Sciences, Lebanese American University, Beirut 1102-2801, Lebanon; 4 Université Libre de Bruxelles, IRIBHM, Brussels B-1070, Belgium; 5 Department of Physical Chemistry, Rudjer Boskovic Institute, 10000 Zagreb, Croatia; 6 Department of Structural Biology, St. Jude Children's Research Hospital, 262 Danny Thomas Place, Memphis, TN 38105, USA

## Abstract

Heterochromatin is a distinctive chromatin structure that is essential for chromosome segregation, genome stability and regulation of gene expression. H3K9 methylation (H3K9me), a hallmark of heterochromatin, is deposited by the Su(var)3-9 family of proteins; however, the mechanism by which H3K9 methyltransferases bind and methylate the nucleosome is poorly understood. In this work we determined the interaction of Clr4, the fission yeast H3K9 methyltransferase, with nucleosomes using nuclear magnetic resonance, biochemical and genetic assays. Our study shows that the Clr4 chromodomain binds the H3K9me3 tail and that both, the chromodomain and the disordered region connecting the chromodomain and the SET domain, bind the nucleosome core. We show that interaction of the disordered region with the nucleosome core is independent of H3K9me and contributes to H3K9me *in vitro* and *in vivo*. Moreover, we show that those interactions with the nucleosome core are contributing to *de novo* deposition of H3K9me and to establishment of heterochromatin.

## INTRODUCTION

Regulation of genome expression is essential for many cellular processes including cell proliferation, differentiation, development and viability. Changes in gene expression and genomic instability are allowing cancer cells to acquire their characteristics. Heterochromatin is one of the distinctive chromatin structures that plays an essential role in chromosome segregation, maintenance of genome stability and regulation of gene expression (1,2). Until last decade, heterochromatin was considered to be transcriptionally silent, but in recent years, it has been shown that heterochromatin is actively transcribed and that transcription is required for heterochromatin formation (3–5).

In fission yeast, siRNAs direct the inactivation of target RNAs by guiding the Argonaute RITS complex to complementary centromeric target sequences (3,6–9). Argonaute recruits the methyltransferase complex CLRC to chromatin, which leads to deposition of repressive histone 3 lysine 9 methylation (H3K9me), a hallmark of heterochromatin (3,4,10,11). The CLRC complex consists of the H3K9 methyltransferase Clr4 and an ubiquitination module that resembles CRL4 type ubiquitin ligase (12–15). Clr4 is a lysine methyltransferase of the CLRC complex that deposits H3K9 methylation on nucleosomes. Subsequently, H3K9 methylation recruits Heterochromatin Protein 1 (HP1) family of proteins and the SHREC complex that mediates transcriptional silencing through histone deacetylation and chromatin-remodeling (2,4,16).

Fission yeast Clr4 is a homologue of the human Su(var)3–9 family of proteins (17). It has an N-terminal chromodomain (CD) and the C–terminal Su(var)3–9 Enhancer of zeste Trithorax (SET) domain (Supplementary Figure S1A) (18,19). The chromodomain and the SET domain are connected by a highly disordered region comprising residues S69-S191. The SET domain consists of several β-strands and loops; and methylates lysine 9 of histone H3 (20). The chromodomain consists of three β-strands and a C-terminal α-helix and specifically binds the H3K9 methylated tail, a product of Clr4 enzymatic activity (21–24). This read/write mechanism is required for heterochromatin maintenance and spreading of heterochromatin beyond initiation sites (25).

Despite the extensive biochemical and genetic studies, the mechanism of H3K9 methylation on the nucleosome remains unclear. Once H3K9 methylation is deposited, the chromodomain will bind the H3K9 methylated tail and tether the SET domain for further methylation steps. How Clr4 is stabilized on the nucleosome during deposition of the initial H3K9 methylation is not understood. In this work we determined the interaction of Clr4 with H3KC9me3 nucleosomes using nuclear magnetic resonance (NMR) Spectroscopy, biochemical and genetic assays. Our study shows that the Clr4 chromodomain binds the H3KC9me3 tail and that both, the chromodomain and the disordered region connecting the chromodomain and the SET domain, bind the nucleosome core. We show that the interaction of the disordered region with the nucleosome core is independent of H3K9 methylation and contributes to H3K9 methylation *in vitro* and *in vivo*. Mutations of Clr4 residues in the disordered region that interact with the nucleosome core reduced binding to the nucleosome and H3K9 methylation *in vitro*. Moreover, our data show that these interactions with the nucleosome core are contributing to *de novo* deposition of H3K9 methylation *in vivo* and to establishment of heterochromatin.

## MATERIALS AND METHODS

### Recombinant protein expression and purification

All Clr4 constructs were generated through inverse polymerase chain reaction (PCR) using the Clr4 full-length plasmid cloned in a pET30a expression vector containing an N-terminal His-tag and C-terminal FLAG-tag (Supplementary Tables S1 and 2). Unlabeled, ^15^N- and ^15^N/^13^C- uniformly labeled Clr4 constructs were all expressed in *Escherichia coli* Bl21(DE3) pLysS cells and purified by affinity chromatography (GE Healthcare) as the following: Protein expression was induced by 0.5 mM Isopropyl β-D-1-thiogalactopyranoside (IPTG) and cell culture was grown for 18 h at 18°C. In the case of ^15^N- and ^15^N/^13^C- Clr4 CD and Clr4 construct 1–191, *E. coli* Bl21(DE3) pLysS cells were grown in 6 liters of M9 minimal medium containing ^15^N-NH_4_Cl and ^13^C-Glucose. Cells were harvested and re-suspended in lysis buffer (50 mM HEPES pH 7.5, 150 mM NaCl, 3 mM beta-mercaptoethanol, 20 mM Imidazole) and flash frozen. Cells were then thawed and incubated for 30 min in lysozyme before sonication (Branson Sonifier 250-output 4, duty cycle 40). After suspension centrifugation at 12 000 *g* for 30 min at 4°C, the supernatant was incubated for 30 min at 4°C with the binding buffer (50 mM HEPES pH 7.5, 500 mM NaCl, 3mM beta-mercaptoethanol, 20 mM Imidazole) on Ni-NTA resin. The protein was eluted from the resin using the elution buffer (50 mM HEPES pH 7.5, 500 mM NaCl, 3 mM beta-mercaptoethanol, 300 mM Imidazole). Clr4 constructs were then dialyzed in a buffer containing 50 mM HEPES pH 7.5, 150 mM NaCl and 3 mM beta-mercaptoethanol. All constructs were further purified by size exclusion chromatography (Superdex 200; GE Healthcare), dialyzed in a buffer containing 50 mM phosphate buffer pH 6.8, 150 mM NaCl, 1 mM Dithiothreitol (DTT) and concentrated by centrifugal filtration.

### Histone protein expression and *in vitro* nucleosome reconstitution

The four *Xenopus laevis* histone proteins H2A, H2B, H3 and H4 were all prepared in a recombinant form after their expression in *E. coli* according to protocol (23,26,27). Lysine 9 of histone H3 (H3K9) was methylated by applying the methyl lysine analog methyl lysine analog (MLA) (23,27). We have confirmed the presence of MLA product with mass spectrometric analysis. The histones were purified in unfolding conditions (6 M Guanidinium Chloride, 20 mM Tris–HCl pH 7.5), mixed to equimolar ratios and dialyzed in refolding buffer (2 M NaCl, 10 mM Tris–HCl pH 7.5) to assemble the octamer. Nucleosomes (methylated, unmodified and tailless) were reconstituted *in vitro* from the octamer and the 167-bp 601 Widom's DNA sequence by salt gradient deposition. The reconstituted nucleosomes were evaluated by agarose, sodium dodecyl sulphate-polyacrylamide gelelectrophoresis (SDS-PAGE) and native gels. To prepare tailless nucleosomes, the reconstituted nucleosomes were incubated for 2 h at 25°C with an immobilized TPCK-Trypsin resin (Thermo Scientific) in a buffer containing 20 mM HEPES pH 7.5, 75 mM NaCl, 1 mM DTT.

### Binding assays and assembly of Clr4–H3KC9 nucleosome complex

The binding assay of all Clr4 constructs and the three types of nucleosomes were conducted as the following: 0.5 μg of protein were bound to 15 μl of anti-FLAG M2 affinity gel resin (Sigma-Aldrich) in binding buffer (50 mM phosphate buffer pH 6.8, 150 mM NaCl, 1 mM DTT) for 20 min at 4°C. The resin was washed once with the binding buffer and 2 mg of nucleosomes were then added to reach a final volume of 40 μl. After 1 h incubation on ice, the resin was centrifuged and the flow-through was collected. The flow-through, resins and inputs were analyzed on SDS-PAGE 15% acrylamide gels then silver staining (0.1% AgNO_3_). All samples were further analyzed with western blot using anti-H3 histone (AbCam, 1:1000), anti-H3K9me3 Antibody (AbCam, 1:1000), anti-goat IgG-HRP (BioRad, 1:3000) and anti-rabbit IgG-HRP (BioRad, 1:3000).

The Clr4-H3KC9me3 nucleosome complexes (Clr4 FL, Clr4 CD and Clr4 1–191 constructs) were prepared as mentioned above using 5 μg protein and 10 μg nucleosomes and eluted after incubation with FLAG peptide (Sigma-Aldrich).

### NMR spectroscopy

All NMR experiments were performed at 15°C on Bruker Avance III 800 MHz spectrometer equipped with a cryogenic probe. Samples of ^15^N/^13^C- Clr4 CD and Clr4 1–191 constructs containing 1 mM protein each were used to run the three-dimensional HNCA, HNCACB, HN(CO)CACB and HNCO triple-resonance experiments (28) for the NMR backbone assignment of Clr4 CD and Clr4 1–191 constructs. The 3D HNCACB and 3D HNCA spectra were recorded with 2048 (F1) × 84 (F2) × 128 (F3) complex points, 24 scans per increment with spectral widths of 7716 Hz, 1705 Hz and 10582 Hz in the ^1^H, ^15^N, ^13^C dimensions, respectively, and a total experiment time of 3 days. The 3D HNCO spectra were recorded with 2048 (F1) × 100 (F2) × 100 (F3) complex points, eight scans per increment with spectral widths of 7003 Hz, 1561 Hz and 1233 Hz in the ^1^H, ^15^N, ^13^C dimensions, respectively, and a total experiment time of 3 days.

The samples used for the NMR titrations contained 50 μM ^15^N-labeled protein (Clr4 CD or Clr4 1–191 construct) in 50 mM phosphate buffer pH 6.8, 150 mM NaCl, 1 mM DTT and 10% (v/v) D_2_O. Different ratios (1:0.5, 1:1 and 1:2) of the Clr4 CD–H3KC9 nucleosome and Clr4 1–191–H3KC9 nucleosome complexes were freshly prepared. Similarly, the [Lys(Me3)9]-Histone H3 (1-21)-GGK(Biotin) peptide (Eurogentec) was used to prepare the ratios (1:1 and 1:5) of the Clr4 CD–H3K9 peptide and Clr4 1–191–H3K9 peptide complexes. The 2D ^1^H-^15^N HSQC experiments were acquired using 600 complex points and 32 scans per increment with spectral widths of 8389 Hz and 1844 Hz in the ^1^H and ^15^N dimensions, respectively, and a total experiment time of 9 h. All NMR experiments were processed with NMRPipe and analyzed with CCPN Analysis (29,30). Averaged chemical shift perturbations were calculated according to the standard equation being ^1^H and ^15^N chemical shifts of free and ligand-bound Clr4 constructs. The intensity ratio plots are reported with a 3-residues averaging window (Supplementary Table S3).

### Clr4 methyltransferase assay

A total of 8 μg of Clr4 and Clr4 MUT12 were mixed with 5 μg of nucleosome, 215 μM SAM (S-adenosyl Methionine) and 36 mM of Tris pH 8. The samples were incubated at 37°C for the time of the reaction. After each time point, SDS loading buffer was added to the tube and the reaction was inactivated at 95°C for 4 min.

For the western blot, the samples were loaded on a 17% SDS gel ran at 300V and the transfer was done at 15V. The membrane was incubated in a 5% blocking buffer for 2 h against H3K9me1 (ab8896, Abcam), H3K9me2 (ab1220, Abcam), H3K9me3 (ab8898, Abcam) or H3 (ab1791, Abcam), then in the first antibody for 1 h. The membrane was then washed with the 1 × TBST buffer. Then it was incubated with the secondary antibody for 1 h. The membrane was washed with the 1 × TBST buffer then mixed with the chemiluminescence substrate Pico plus for the revelation.

### Strain construction, plasmid generation and genomic integration of point mutants

The strains were constructed by electroporation (Biorad MicroPulser program ShS) with a PCR-based gene targeting product leading to deletion or epitope-tagging of specific genes (Supplementary Tables S1 and 2). Plasmids were cloned by enzyme digestion and subsequent DNA-ligation. Mutations were introduced with inverse PCR (31). For genomic integration of the mutants, a PCR with long overhang primers according to Bähler *et al.* (32) was performed and the product transformed. Positive transformants were selected on YES plates containing 100–200 mg/ml antibiotics and were confirmed by PCR and sequencing.

### Growth assay and *ade6* reporter spot assay

Tenfold serial dilutions of cultures with OD_600_ between 0.7 and 1.5 were made so that the highest density spot contained 10^5^ cells. Cells were spotted on non-selective YES medium or not supplemented YE (low adenine) medium. The plates were incubated at 32°C for 2–3 days and imaged. Cells with a silenced *ade6* gene are red, cells expressing *ade6* are white. In pink colonies the ade6 gene is partially de-repressed. Trichostatin (TSA) treated cells were grown in 35 mg/ml TSA for 24 h and then plated on low adenine YE plates.

### *In vitro* Clr4 activity assay with RNA inhibition

In order to determine the inhibitory effect of RNA on Clr4 activity, a nucleosome methylation assay was performed. Each reaction contained 3 μM Clr4 and 30 nM nucleosomes in 1× reaction buffer (0.1 M Tris pH 8.8, 0.1 M KCl, 5% (v/v) glycerol, 1 mM MgCl_2_, 20 μM ZnSO_4_, 10 mM ß-Mercaptoethanol). Furthermore, 80 μM SAM and different concentrations of 100 nt + 20A RNA (15 nM – 1.5 μM in 10 mM Tris) were added. After 5 h incubation at 30°C, Clr4 was inactivated at 95°C for 3 min. RNA was degraded with 1 μl RNase A for 5 min at RT. The activity was evaluated by 15% acrylamide SDS-page and western blot analysis using antibodies against H3K9me2 (ab1220, Abcam) and H3 (ab1791, Abcam).

## RESULTS

### Disordered region of Clr4 binds the nucleosome independently of H3K9 methylation

**Figure 1.**
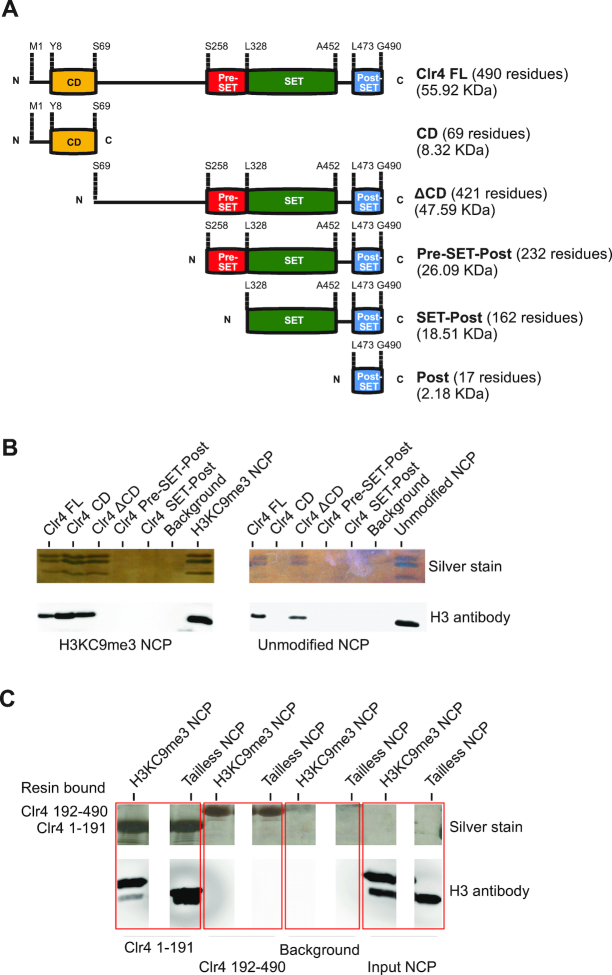
Assembly of the Clr4–H3KC9 methylated nucleosome complex. (**A**) A scheme showing the domain organization of the full-length Clr4 protein (FL) and the designed constructs. N = N-terminal, C = C-terminal, CD = chromodomain, SET = SET domain. (**B**) Silver stained SDS-PAGE and western blot analysis showing interaction of indicated Clr4 constructs with H3KC9me3 and unmodified nucleosomes. Clr4 constructs were bound to FLAG resin and incubated with nucleosomes. Elutions from the resin are shown. (**C**) Silver stained SDS-PAGE and western blot analysis of resin bound FLAG-tagged Clr4 constructs Clr4_1–191 and Clr4_192–491 and co-purifying H3KC9me3 and tailless nucleosomes. Clr4 constructs were bound to FLAG resin and incubated with nucleosomes. Elutions from the resin are shown.

To study Clr4 interaction with the nucleosome, we expressed and purified full-length Clr4, Clr4 chromodomain (Clr4 CD) and a construct that lacks the chromodomain (Clr4 ΔCD) (Figure 1A and Supplementary Figure S1A). The Clr4 ΔCD construct was further divided into the Pre-SET-Post construct that lacks the disordered region and the SET-Post construct that lacks the disordered region and the Pre-SET domain (Figure 1A and Supplementary Figure S1A). The histone proteins H2A, H2B, H3 and H4 were expressed and purified and nucleosomes were reconstituted as described (26,33) (Supplementary Figure S1B and C). The H3K9me3 nucleosomes were assembled using the MLA method (H3KC9me3 analog) and were confirmed by mass spectrometric analysis as previously described (Supplementary Figure S1B–D) (23,34).

Next, we tested binding of full length and mutant Clr4 constructs to nucleosomes which were either methylated on H3K9 (H3KC9me3), unmodified or did not have histone tails (tailless) (Figure 1B). In the pull-down assays, the C-terminal FLAG-tag containing constructs were bound to the anti-FLAG resin and the interaction with the nucleosomes was probed. Unbound nucleosomes were washed away and binding was determined by gel electrophoresis. Our data show that the full-length Clr4 and Clr4 ΔCD bind both, H3KC9me3 and unmodified nucleosomes (Figure 1B). The Clr4 CD construct, however, binds only the H3KC9me3, but not the unmodified nucleosome (Figure 1B). This indicates that the chromodomain requires the H3KC9me3 tail for interaction with the nucleosome, while full-length Clr4 and Clr4 ΔCD can also bind the nucleosome independent of H3K9 methylation. We have also observed that Clr4 and Clr4 ΔCD constructs bind tailless nucleosomes, indicating that these constructs bind the core of the nucleosome (Supplementary Figure S1E). The two other constructs (Pre-SET-Post and SET-Post) did not bind to the nucleosomes suggesting either no binding or a very transient binding, which is not stable enough to be detected in the pull-down assays (Figure 1B). Our data show that the Clr4 ΔCD construct (residues 69–490) binds unmodified and tailless nucleosomes, while the Pre-SET-Post construct (residues 258–490) shows no binding to any kind of nucleosomes. This indicates that chromodomain and disordered region are required for the SET domain to bind and methylate the nucleosome. Notably, our data show that interaction with the nucleosome core is not specific to H3K9 methylation and is mediated by the disordered region connecting the chromodomain and the SET domain (Figure 1B).

To further dissect the role of the disordered region in binding to the nucleosome, we generated Clr4 constructs comprising residues 1–191 (Clr4_1–191 which includes CD and the disordered region) and 192–490 (Clr4_192–460 which includes the SET domain). The pull-down assays revealed that the Clr4_1–191 construct, but not Clr4_192–490, binds H3KC9me3 and tailless nucleosomes (Figure 1C). These data show that Clr4 can bind the nucleosome independent of H3K9 methylation through the highly disordered region comprising residues 70–191.

### Clr4 binds H3KC9me3 nucleosome through the chromodomain and the disordered region

**Figure 2.**
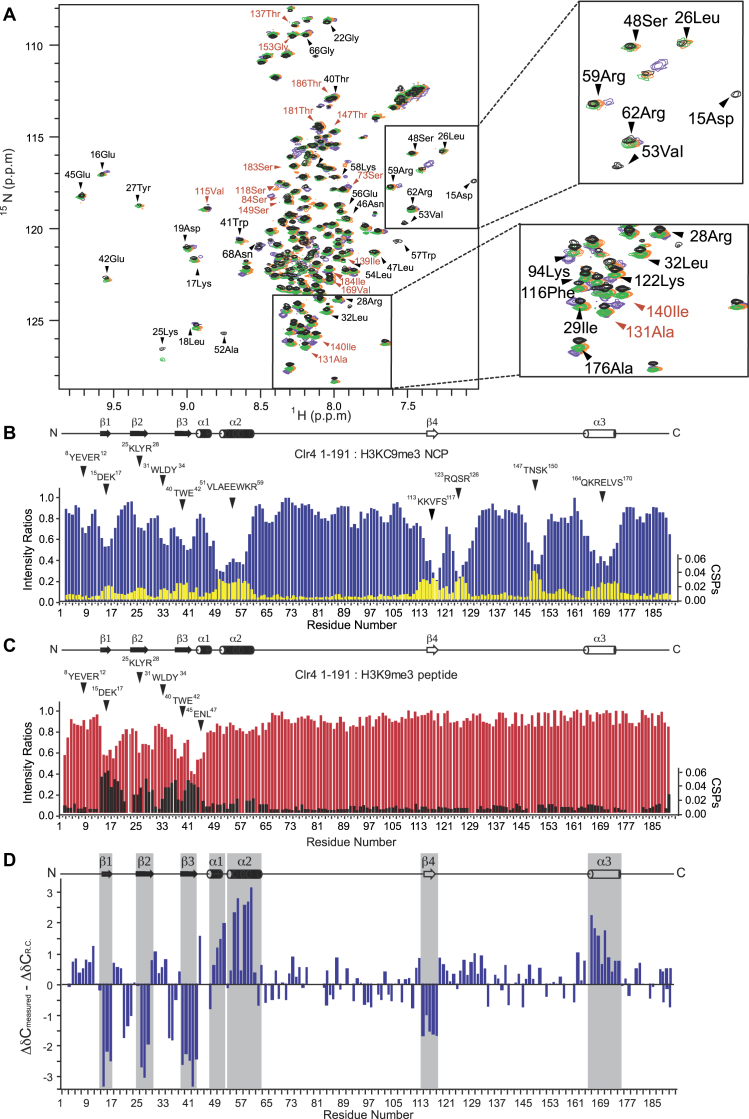
The interaction between Clr4 construct 1–191 and the H3KC9me3 nucleosome. (**A**) Two-dimensional ^1^H-^15^N HSQC spectra of the isotope-labeled Clr4_1–191 construct in the absence (black) and presence of increased ratios of unlabeled H3KC9me3 nucleosome; Clr4_1–191:H3KC9me3 nucleosome (1:0.5 green, 1:1 orange, 1:2 purple). The selected chemical assignments highlight perturbation and broadening in the chemical shifts of certain resonances. (**B**) Intensity ratio plot (blue) and chemical shifts perturbations CSPs (yellow) of Clr4_1–191 in the presence of the H3KC9me3 nucleosome (Clr4_1–191:H3KC9me3 nucleosome 1:2). Intensity ratios and weighted chemical shift perturbations correspond to the backbone amide resonances. The residue patches of the chromodomain (^15^DEK^17^, ^25^KLYR^28^, ^40^TWE^42^) showed most significant perturbations and decrease in signal intensities in presence of both peptide and nucleosomes. Signal broadening and perturbations were detected for the residue patches in the unfolded region only in the case when bound to the nucleosome and not to the H3K9 peptide. (**C**) Intensity ratio plot (red) and chemical shifts perturbations CSPs (black) of Clr4_1–191 in the presence of the H3K9 methylated peptide (Clr4_1–191:H3K9me3 Peptide 1:5). (**D**) Secondary structure elements of Clr4 1–191 bound to the H3KC9me3 nucleosome. The indicated secondary structure is based on the Cα NMR chemical shifts analysis and represents differences between measured Cα and random coil (RC) chemical shifts as a function of the primary sequence in Clr4. The secondary structure elements are highlighted in gray. Cα atoms in α-helices and β-sheets have positive and negative secondary chemical shifts, respectively.

To analyze the Clr4 interaction with the nucleosome we used NMR spectroscopy. We acquired 2D heteronuclear single quantum coherence (HSQC) spectra of ^15^N-labeled Clr4 CD in its free form, bound to the H3K9me3 peptide, to the H3KC9me3 nucleosome, to the unmodified nucleosome and to DNA (Figure 2 and Supplementary Figure S2). This method allows deciphering the interaction sites by recording the changes in chemical shifts and intensities of individual peaks. The superposition of 2D ^1^H-^15^N HSQC spectra of free and bound forms of the protein directly indicates which residues change their chemical shift positions and signal intensities. The analysis of the NMR intensity ratios I/I_0_ (I is the intensity of bound form, I_0_ is the intensity of free form) with respect to the residue number of the protein is a direct indication of binding. Signal broadening (I/I_0_ < 1) is due to exchange of the protein between the free and bound conformations. The reduction in the signal intensity is a common observation in protein NMR spectroscopy that reflects the combination of the molecular weight increase upon complex formation and the chemical exchange at the contact surface (35).

First, we investigated the binding of Clr4 CD to thDuring titration ofe H3K9me3 peptide using NMR spectroscopy. Upon Clr4 CD binding to H3K9me3 peptide, a subset of signals shifted and strongly decreased in intensities. The overlap of 2D ^1^H-^15^N NMR HSQC spectra between the apo and H3K9me3 peptide_bound Clr4 CD confirms that the Clr4 CD structure remains unaltered after binding to the H3K9me3 peptide (Supplementary Figure S2A). The NMR data show that the Clr4 CD binding to the H3K9me3 peptide involved patches ^15^DEK^17^, ^25^KLYR^28,40^TWE^42^ and ^45^ENL^47^ (Supplementary Figure S2B and C), which is consistent with the structure of the Clr4 chromodomain (Supplementary Figure S2C) (24). In agreement with previous structures (21,22,24), our data show that the conserved aromatic cage binds the H3K9me3 tail. To determine if Clr4 CD might bind the core of the nucleosome, we investigated Clr4 CD interaction with the H3KC9me3 nucleosome. The interaction with the nucleosome involved residue patches ^8^YEVER^12^, ^15^DEK^17^, ^25^KLYR^28^, ^31^WLNY^34^, ^40^TWE^42^ and ^56^EWKR^59^ (Supplementary Figure S2D and E). The last ^56^EWKR^59^ patch is in the C-terminal helix of the chromodomain, and our data show that these residues interact only with the H3KC9me3 nucleosome and not with the H3K9me3 peptide (Figure 2A–C; Supplementary Figure S2D and E). This is in agreement with crystal structures of different chromodomains where the interaction between the C-terminal helix and the H3K9me3 peptide was also not observed (21,22,24).

The chromodomain consists of a three-stranded antiparallel β-sheet, a long C-terminal α-helix and a disordered loop that separates β2 and β3 strands. Based on the strong correlation between chemical shifts and local structure, we derived the secondary chemical shifts for the Clr4 CD bound to the H3KC9me3 nucleosome to confirm the secondary structure elements (Supplementary Figure S3A and B). This is based on the difference between the measured Cα chemical shifts and random coil chemical shifts as a function of the primary sequence of Clr4 CD. Cα atoms in α-helices and β-sheets will have positive and negative secondary chemical shifts, respectively. Our data show that the secondary structure elements remain unaltered after binding the H3KC9me3 nucleosome and are consistent with the solution structure (24).

Our binding assays show that the disordered region between residues 69 and 191 contributes to nucleosome binding independent of H3K9 methylation (Figure 1B). This suggests that the disordered region makes additional contact sites with the nucleosome that might stabilize the complex. We used NMR to determine the interaction between the disordered region and the H3KC9me3 nucleosome, unmodified nucleosome and DNA. We observed changes in NMR signal position and intensity in 2D ^1^H-^15^N HSQC spectra after binding of the Clr4_1–191 construct to the H3KC9me3 nucleosome. Similar to the binding of Clr4 CD, this included the residue patches ^8^YEVER^12^, ^15^DEK^17^, ^25^KLYR^28^, ^31^WLDY^34^, ^40^TWE^42^ and ^51^VLAEEWKR^59^ (Figure 2A–C). These patches show the interaction of Clr4 chromodomain with the H3KC9me3 tail and the core of the nucleosome. The interaction of the C-terminal α-helix of the chromodomain with the H3KC9me3 nucleosome is more extensive when the disordered region is present (Figure 2B and Supplementary Figure S2D), suggesting that the disordered region stabilizes the CD interaction with the core of the nucleosome (Supplementary Figure S4A and B). Our NMR data show that CD in Clr4 1_191 also binds the unmodified nucleosome, however interactions involved in binding the H3KC9me3 residue are lost (Supplementary Figure S5A–C). Notably, we observed that residues K58-R62 of the C-terminal helix bind only H3KC9me3 nucleosome and not the unmodified nucleosome or DNA, indicating that this interaction is H3K9me3 dependent (Figure 2 and Supplementary Figure S5A–F). Altogether, our data show that the disordered region tethers Clr4 CD to the nucleosome, and that CD interacts with the unmodified nucleosome in a similar way as with the H3KC9me3 nucleosome.

Moreover, we observe several interactions of the disordered region with the H3KC9me3 nucleosome. This includes the patches ^113^KKVFS^117^, ^123^RQSR^126^, ^147^TNSK^150^ and ^164^QKRELVS^170^ in the disordered region. The secondary chemical shifts of the Clr4_1–191 construct bound to the H3KC9me3 nucleosome confirmed that the three-stranded anti-parallel β-sheets and the two α-helices all remain intact at the CD domain (Figure 2D and Supplementary Figure S6A). Notably, the propensity for secondary structure elements was visible at the intrinsically disordered region upon Clr4_1–191 binding to the H3KC9me3 nucleosome. The patch ^113^KKVFS^117^ incorporates the β-strand and ^164^QKRELVS^174^ shows features of an α-helix (Figure 2D). These structural elements were not visible when Clr4_1–191 was bound to the H3K9me3 peptide (Supplementary Figure S6A). Our data show that Clr4 binds the nucleosome with its disordered region independent of H3K9 methylation and this induces formation of an α-helix and β-sheet in this region. The interaction of the disordered region with the nucleosome also stabilizes the chromodomain interaction.

We observed that in NMR spectra showing interaction of Clr4 1–191 with H3KC9me3 nucleosome, a subset of peaks changes chemical shifts in a linear fashion (59 Arg, 94 Lys, 16 Glu, 19 Asp), while others change chemical shifts in a non-linear fashion (147 Tyr, 40 Thr, 22 Gly, 42 Glu, 29 Ile and 176 Ala) (Figure 2A). Moreover, at higher concentrations, some peaks are broadened beyond detection (53 Val, 15 Asp, 52 Ala). For some of these residues, a two-state interaction cannot explain the deviation from linearity while a complex binding interaction exists. During titration of the ligand (Clr4) to the complex (nucleosome) broadening and perturbation of chemical shifts occur due to (i) intermediate to slow exchange between free and bound states, (ii) structural re-arrangement of protein/ligand complex after binding or (iii) conformational change of the protein complex prior ligand binding. All these interactions most likely lead to peaks in 2D spectra to move in a non-linear fashion while going from free to bound forms. The deviation from linearity will depend on the populations of intermediate states. Non-linear behavior can also be caused by factors other than two-state binding interaction such as multiple bindings where a strong binding occurs at a specific site together with a weak non-specific binding at multiple sites (36). The residues in Clr4 showing non-linear behavior are most likely affected by other factors like electrostatic interaction of DNA with charged residues and conformational exchange at increasing nucleosome concentration. Given the dynamic histone–DNA interactions (27,33) and possible presence of free DNA (Supplementary Figure S1C), deviation from a linearity may arise due to interaction with multiple species.

Consistent with the binding assays (Figure 1B and C), our NMR data show that the disordered region binds the unmodified nucleosome and DNA (Supplementary Figure S6B). Most residues in the disordered region bind the H3KC9me3 nucleosome, the unmodified nucleosome and DNA, indicating that these residues bind primarily nucleosomal DNA. Only the patch ^164^QKRELVS^174^ does not bind the unmodified nucleosome, and these residues interact with the nucleosome in an H3K9me3 dependent manner and fold into an α-helix (Figure 2B and Supplementary Figure S6B).

### Clr4 disordered region contributes to H3K9 methylation and heterochromatin establishment

**Figure 3.**
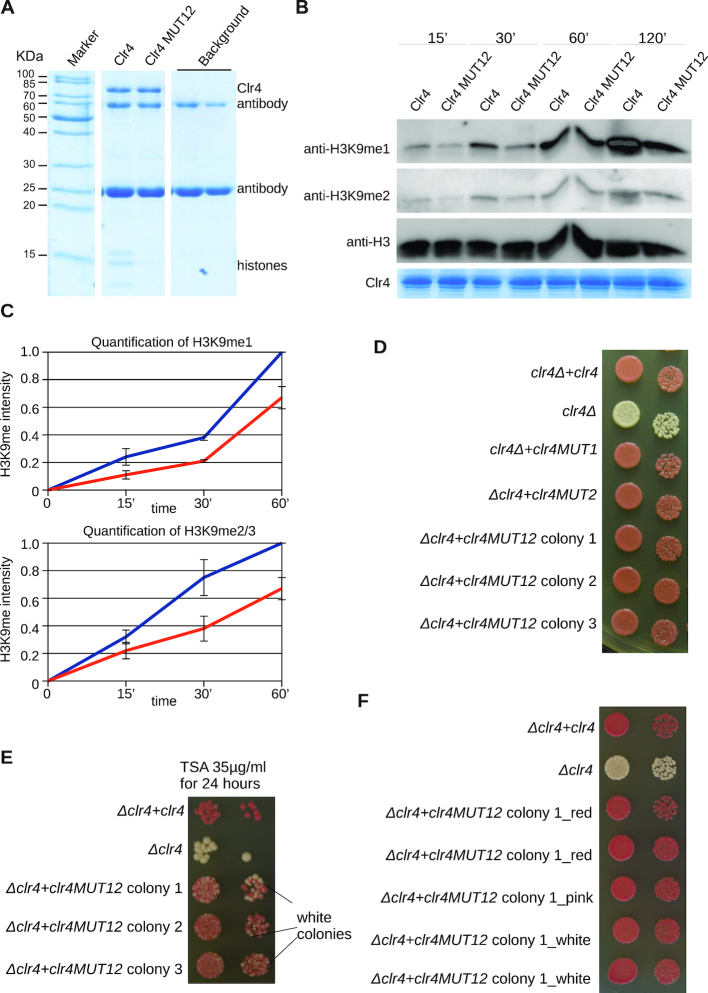
Interaction of Clr4 disordered region with the nucleosome promotes H3K9 methylation and heterochromatin establishment. (**A**) SDS-PAGE analysis of resin bound FLAG-tagged wild-type and MUT12 Clr4 constructs and co-purifying unmodified nucleosomes. Clr4 MUT12 has weaker affinity for the nucleosome. (**B**) *In vitro* assay showing H3K9 methylation by wild-type and mutant Clr4 (MUT12). Clr4 was incubated with the nucleosomes in presence of SAM and reaction was stopped at indicated time points. H3K9 methylation was detected by western blot. (**C**) Quantification of Clr4 activity assay western blots. For H3K9me1 two independent assays were averaged, and for H3K9me2/3 four assays were averaged. The assays were quantified with Quantity One with local background subtraction. Standard error for each data point is shown. (**D**) Growth assay with centromeric *ade6* reporter gene showing that mutations in the disordered region of Clr4 do not have an effect on heterochromatin maintenance. Red colonies indicate silencing of *ade6* gene and formation of functional heterochromatin. Cells were plated in 10-fold dilutions starting with 10^5^ cells. (**E**) Growth assay with centromeric *ade6* reporter gene after heterochromatin perturbation with deacetylase inhibitor TSA. Heterochromatin establishment is perturbed in cells having mutations in Clr4 disordered region. Appearance of white colonies indicates defect in heterochromatin formation and loss of *ade6* silencing. (**F**) Growth assay with centromeric *ade6* reporter gene. White colonies from (B) were grown for 2 days and plated. All white colonies turned red, indicating that they established functional heterochromatin.

Next, we tested if the interaction of the Clr4 disordered region with the nucleosome contributes to H3K9 methylation and heterochromatin formation in fission yeast cells. We mutated two patches in the Clr4 disordered region that make contacts with the nucleosome in our NMR data. The patch ^164^QKRELVS^170^ in the *clr4* gene was mutated to ^164^SGSGSGS^170^ and the patch ^147^TNSK^150^ to ^147^SGSG^150^, respectively, generating the MUT12 construct. We purified wild-type and mutant Clr4 and determined their methlytransferase activity by western blot (Supplementary Figure S7A). Our biochemical data show that mutations in these two patches reduced Clr4 interaction with the nucleosome and the methyltransferase activity (Figure 3A–C). This indicates that interaction of the disordered region with the nucleosome stabilizes Clr4 on the nucleosome and promotes H3K9 methylation.

We mutated the same patches in the *clr4* gene and inserted the mutant *clr4* into the genome of a *clr4* deletion strain. This generated fission yeast strains MUT1 (^164^SGSGSGS^170^) and MUT2 (^147^SGSG^150^) with mutations in the *clr4* disordered region integrated into the genome. We also generated the MUT12 strain that has mutations in both patches. To exclude variation in the expression due to genomic manipulation, we re-integrated the wild-type *clr4* gene as a control. These mutations were inserted in a strain containing an *ade6* reporter gene in the pericentromeric heterochromatin. When grown on low adenine medium, cells that silence the *ade6* reporter gene will be red, and cells that express *ade6* will be white. We have grown our mutant and control strains on a low adenine medium YE and observed only red colonies, comparable to wild-type cells (Figure 3D). This indicates that in our mutant strain the *ade6* reporter is silenced and that heterochromatin formation is not impaired when the disordered region of *clr4* is mutated, although the methyltransferase activity is reduced. Our data show that interaction of Clr4 disordered region with the nucleosome is not required for heterochromatin maintenance.

To test if this interaction might be required for establishment of heterochromatic silencing, we perturbed heterochromatin by adding TSA to the media. TSA inhibits deacetylases and interferes with heterochromatin formation (7,37). Cells were grown on 35 μg/ml of TSA for 24 h and then directly plated on a low adenine YE medium. Under these conditions, we observed a higher percentage of white colonies in our MUT12 strains (Figure 3E), indicating that some cells did not efficiently re-establish heterochromatin after perturbation. The control cells efficiently re-established pericentromeric heterochromatin. These data show that interaction of the disordered region of Clr4 with the nucleosome contributes to heterochromatin establishment.

Next, we took several single white MUT12 colonies that did not establish heterochromatin in our establishment assay. We have grown these colonies for 2 days and plated them again on low adenine YE medium to determine if these cells eventually succeed in establishing heterochromatin. Our data show that every single white colony lead to a red progeny that is efficiently silencing the *ade6* reporter gene (Figure 3F). Eventually, the mutant cells succeeded in establishing functional heterochromatin, however, this was less efficient and slower than in wild-type cells. This indicates that the interaction of Clr4 disordered region with the nucleosome increases efficiency and the kinetics of H3K9 methylation and heterochromatin establishment.

Taken together, our data show that in absence of H3K9 methylation, Clr4 disordered region binds the nucleosome which stabilizes the complex to deposit the initial H3K9 methylation. This interaction increases the efficiency of H3K9 methylation and heterochromatin establishment. Once initial H3K9 methylation is deposited, Clr4 chromodomain can bind the H3K9 methylated nucleosomes which will tether Clr4 for subsequent methylation steps.

### Clr4 methyltransferase activity is inhibited by RNA

Our recent data show that RNA accumulation on chromatin interferes with H3K9 methylation and heterochromatin formation (38). The mechanisms of the RNA mediated loss of H3K9 methylation and heterochromatin remained; however, unclear. Here, we investigated if RNA might inhibit Clr4 methyltransferase activity on the nucleosome which will lead to loss of H3K9 methylation and heterochromatin formation.

**Figure 4.**
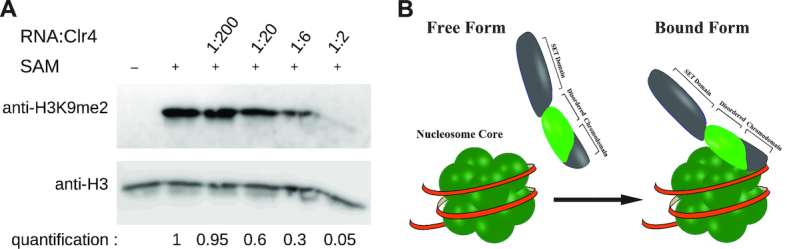
RNA inhibits H3K9 methylation by Clr4. (**A**) *In vitro* assay showing H3K9 methylation by wild-type Clr4 in presence of increasing amounts of RNA. H3K9 methylation was detected by western blot. Increasing amounts of RNA inhibit H3K9 methylation by Clr4. (**B**) Clr4 binds unmodified nucleosomes with its disordered region. The disordered region folds on the nucleosome and stabilizes the complex. This promotes H3K9 methylation by Clr4 and heterochromatin establishment.

Clr4 interaction with nucleic acids has been proposed (39), however, it remains unclear how this interaction regulates its activity. Clr4 chromodomain residues K58-R62 were shown to be essential for interaction with RNA or DNA and our NMR data show that the same residues bind the H3KC9me3 nucleosome, most likely nucleosomal DNA (Figure 2B). To test the possibility that RNA might interfere with Clr4 activity, we added RNA to our *in vitro* methlytransferase activity assay and observed that RNA strongly inhibits H3K9 methylation of nucleosomes (Figure 4A). This suggests that interaction with RNA prevents Clr4 interaction with the nucleosomal DNA, which reduces Clr4 methyltransferase activity.

## DISCUSSION

The organization of chromatin into heterochromatin is essential for epigenetic silencing and chromosome segregation. H3K9 methylation is a hallmark of heterochromatin, however, the mechanisms of its deposition are still poorly understood. In this work we have determined the interaction of Clr4 with the nucleosomes using NMR spectroscopy, pull-down assays and *in vivo* heterochromatin establishment assays. Our data show that Clr4 interaction with the nucleosome is mediated by the chromodomain and the disordered region connecting chromodomain and the SET domain. Chromodomain binds only nucleosomes with methylated H3K9, while the disordered region binds both methylated and unmodified nucleosomes. These data reveal an unexpected role for the disordered region in a non-specific interaction with the nucleosome that is important for *de novo* H3K9 methylation and heterochromatin establishment (Figure 4B). Our data suggest that this interaction, which is independent of H3K9 methylation, stabilizes Clr4 on the unmodified nucleosomes to deposit the first H3K9 methylation mark and to establish a silent chromatin state. After initial H3K9 methylation is deposited, the chromodomain will bind it and tether Clr4 to the nucleosome to deposit additional H3K9 methylation. This read/write mechanism is essential for heterochromatin formation and its spreading beyond the nucleation sites (25).

Analysis of NMR secondary chemical shifts confirmed that the secondary structure elements in the chromodomain remain unaltered after binding to the nucleosome. Notably, we found the propensity for secondary structure elements in the intrinsically disordered region between S69 and S192 with β-sheet β_4_ (^113^KKVFS^117^) and α-helix α_3_ (^164^QKRELVS^174^). These data show that the nucleosome induces formation of secondary structure elements in the disordered region of Clr4 and these elements bind the core of the nucleosome (40–42).

Moreover, we observe that the C-terminal helix of the Clr4 chromodomain interacts with the H3KC9me3 nucleosome, but not with the H3K9me3 peptide. This suggests that the C-terminal helix of Clr4 binds the core of the nucleosome. Previous results show that the Clr4 CD can bind RNA or DNA with its positively charged residues K58-R62 in the C-terminal helix in the H3K9me3-dependent manner (39). Consistent with previous results, we do not observe interaction of these residues with the unmodified nucleosome or DNA in our NMR data, neither we do observe Clr4 CD binding to the unmodified nucleosome. Notably, several patches in CD bind the unmodified nucleosome in an H3K9me3 independent way, but this interaction is dependent on the disordered region. This indicates that the disordered region recruits CD to the unmodified nucleosome and that CD binds unmodified and H3K9me3 nucleosome in a similar way.

Our data show that Clr4 CD and the disordered region bind nucleosomal DNA. This suggests that RNA might compete with Clr4 binding to the nucleosome, which might inhibit Clr4 methyltransferase activity. We have tested this possibility and observed that presence of RNA strongly inhibits Clr4 methyltransferase activity *in vitro*. This is in agreement with our recent work which shows that chromatin bound RNA inhibit heterochromatin formation in fission yeast cells (38). These data suggest that chromatin bound RNA binds the Clr4 chromodomain and disordered region which prevents its interaction with the nucleosome and reduces H3K9 methyltransferase activity. In agreement, recent data show that RNA inhibits activity of Polycomb Repressive Complex 2 (PRC2) which methylates H3K27, another repressive histone mark (43,44).

Our data suggest that the interaction of the disordered region with the nucleosome promotes establishment of H3K9 methylation. This might have an implication on establishment of ectopic H3K9 methylation in human cancer cells. G9a, a mammalian H3K9 methyltransferase, was shown to deposit ectopic H3K9 methylation in many cancers, including breast and lung cancers (45,46). The H3K9 methyltransferase activity of G9a was shown to be important for cancer proliferation, underlining the importance in understanding how the initial H3K9 methylation is deposited. Our data suggest that disordered regions of histone methyltransferases contribute to the binding to unmodified nucleosomes for deposition of the first methylation mark. This might lead to propagation of ectopic H3K9 methylation, cell reprogramming and formation of cancer cells.

## DATA AVAILABILITY

The NMR data are deposited in BMRB under accession number 27903.

## Supplementary Material

gkz480_Supplemental_File

## References

[1] Heard E., Martienssen R.A. Transgenerational epigenetic inheritance: myths and mechanisms. Cell. 2014; 157:95–109. 24679529 10.1016/j.cell.2014.02.045 PMC4020004

[2] Moazed D. Mechanisms for the inheritance of chromatin states. Cell. 2011; 146:510–518. 21854979 10.1016/j.cell.2011.07.013 PMC3244757

[3] Holoch D., Moazed D. RNA-mediated epigenetic regulation of gene expression. Nat. Rev. Genet. 2015; 16:71–84. 25554358 10.1038/nrg3863 PMC4376354

[4] Allshire R.C., Ekwall K. Epigenetic regulation of chromatin states in Schizosaccharomyces pombe. Cold Spring Harb. Perspect. Biol. 2015; 7:a018770. 26134317 10.1101/cshperspect.a018770 PMC4484966

[5] Martienssen R., Moazed D. RNAi and heterochromatin assembly. Cold Spring Harb. Perspect. Biol. 2015; 7:a019323. 26238358 10.1101/cshperspect.a019323 PMC4526745

[6] Halic M., Moazed D. Dicer-independent primal RNAs trigger RNAi and heterochromatin formation. Cell. 2010; 140:504–516. 20178743 10.1016/j.cell.2010.01.019 PMC3020400

[7] Marasovic M., Zocco M., Halic M. Argonaute and Triman generate dicer-independent priRNAs and mature siRNAs to initiate heterochromatin formation. Mol. Cell. 2013; 52:173–183. 24095277 10.1016/j.molcel.2013.08.046

[8] Pisacane P, Halic M Tailing and degradation of Argonaute-bound small RNAs protect the genome from uncontrolled RNAi. Nat.Commun. 2017; 8:15332–15345. 28541282 10.1038/ncomms15332 PMC5458512

[9] Castel S.E., Martienssen R.A. RNA interference in the nucleus: roles for small RNAs in transcription, epigenetics and beyond. Nat. Rev. Genet. 2013; 14:100–112. 23329111 10.1038/nrg3355 PMC4205957

[10] Volpe T.A., Kidner C., Hall I.M., Teng G., Grewal S.I.S., Martienssen R.A. Regulation of heterochromatic silencing and histone H3 lysine-9 methylation by RNAi. Science. 2002; 297:1833–1837. 12193640 10.1126/science.1074973

[11] Verdel A., Jia S., Gerber S., Sugiyama T., Gygi S., Grewal S.I.S., Moazed D. RNAi-mediated targeting of heterochromatin by the RITS complex. Science. 2004; 303:672–676. 14704433 10.1126/science.1093686 PMC3244756

[12] Horn P.J., Bastie J.-N., Peterson C.L. A Rik1-associated, cullin-dependent E3 ubiquitin ligase is essential for heterochromatin formation. Genes Dev. 2005; 19:1705–1714. 16024659 10.1101/gad.1328005 PMC1176008

[13] Jia S., Kobayashi R., Grewal S.I.S. Ubiquitin ligase component Cul4 associates with Clr4 histone methyltransferase to assemble heterochromatin. Nat. Cell Biol. 2005; 7:1007–1013. 16127433 10.1038/ncb1300

[14] Hong E.-J.E., Villén J., Gerace E.L., Gygi S.P., Moazed D. A cullin E3 ubiquitin ligase complex associates with Rik1 and the Clr4 histone H3-K9 methyltransferase and is required for RNAi-mediated heterochromatin formation. RNA Biol. 2005; 2:106–111. 17114925 10.4161/rna.2.3.2131

[15] Kuscu C., Zaratiegui M., Kim H.S., Wah D.A., Martienssen R.A., Schalch T., Joshua-Tor L. CRL4-like Clr4 complex in Schizosaccharomyces pombe depends on an exposed surface of Dos1 for heterochromatin silencing. Proc. Natl. Acad. Sci. U.S.A. 2014; 111:1795–1800. 24449894 10.1073/pnas.1313096111 PMC3918804

[16] Grewal S.I. RNAi-dependent formation of heterochromatin and its diverse functions. Curr. Opin. Genet. Dev. 2010; 20:134–141. 20207534 10.1016/j.gde.2010.02.003 PMC3005588

[17] Lejeune E., Allshire R.C. Common ground: small RNA programming and chromatin modifications. Curr. Opin. Cell Biol. 2011; 23:258–265. 21478005 10.1016/j.ceb.2011.03.005

[18] Fodor B.D., Shukeir N., Reuter G., Jenuwein T. Mammalian Su(var) genes in chromatin control. Annu. Rev. Cell Dev. Biol. 2010; 26:471–501. 19575672 10.1146/annurev.cellbio.042308.113225

[19] Allis C.D., Jenuwein T. The molecular hallmarks of epigenetic control. Nat. Rev. Genet. 2016; 17:487–500. 27346641 10.1038/nrg.2016.59

[20] Min J., Zhang X., Cheng X., Grewal S.I.S., Xu R.-M. Structure of the SET domain histone lysine methyltransferase Clr4. Nat. Struct. Biol. 2002; 9:828–832. 12389037 10.1038/nsb860

[21] Jacobs S.A., Khorasanizadeh S. Structure of HP1 chromodomain bound to a lysine 9-methylated histone H3 tail. Science. 2002; 295:2080–2083. 11859155 10.1126/science.1069473

[22] Nielsen P.R., Nietlispach D., Mott H.R., Callaghan J., Bannister A., Kouzarides T., Murzin A.G., Murzina N.V., Laue E.D. Structure of the HP1 chromodomain bound to histone H3 methylated at lysine 9. Nature. 2002; 416:103–107. 11882902 10.1038/nature722

[23] Zocco M., Marasovic M., Pisacane P., Bilokapic S., Halic M. The Chp1 chromodomain binds the H3K9me tail and the nucleosome core to assemble heterochromatin. Cell Discov. 2016; 2:16004–16019. 27462451 10.1038/celldisc.2016.4 PMC4849473

[24] Horita D.A., Ivanova A.V., Altieri A.S., Klar A.J., Byrd R.A. Solution structure, domain features, and structural implications of mutants of the chromo domain from the fission yeast histone methyltransferase Clr4. J. Mol. Biol. 2001; 307:861–870. 11273706 10.1006/jmbi.2001.4515

[25] Zhang K., Mosch K., Fischle W., Grewal S.I.S. Roles of the Clr4 methyltransferase complex in nucleation, spreading and maintenance of heterochromatin. Nat. Struct. Mol. Biol. 2008; 15:381–388. 18345014 10.1038/nsmb.1406

[26] Ivic N, Groschup B, Bilokapic S, Halic M Simplified method for rapid purification of soluble histones. Croat. Chem. Acta. 2016; 89:153–162.

[27] Bilokapic S., Strauss M., Halic M. Structural rearrangements of the histone octamer translocate DNA. Nat.Commun. 2018; 9:1330–1341. 29626188 10.1038/s41467-018-03677-z PMC5889399

[28] Anglister J., Grzesiek S., Ren H., Klee C.B., Bax A. Isotope-edited multidimensional NMR of calcineurin B in the presence of the non-deuterated detergent CHAPS. J. Biomol. NMR. 1993; 3:121–126. 8383554 10.1007/BF00242480

[29] Vranken W.F., Boucher W., Stevens T.J., Fogh R.H., Pajon A., Llinas M., Ulrich E.L., Markley J.L., Ionides J., Laue E.D. The CCPN data model for NMR spectroscopy: development of a software pipeline. Proteins. 2005; 59:687–696. 15815974 10.1002/prot.20449

[30] Delaglio F., Grzesiek S., Vuister G.W., Zhu G., Pfeifer J., Bax A. NMRPipe: a multidimensional spectral processing system based on UNIX pipes. J. Biomol. NMR. 1995; 6:277–293. 8520220 10.1007/BF00197809

[31] Ulrich A., Andersen K.R., Schwartz T.U. Exponential megapriming PCR (EMP) cloning–seamless DNA insertion into any target plasmid without sequence constraints. PLoS One. 2012; 7:e53360. 23300917 10.1371/journal.pone.0053360 PMC3534072

[32] Bähler J., Wu J.Q., Longtine M.S., Shah N.G., McKenzie 3rd, Steever A., Wach A.B., Philippsen A., Pringle J.R. Heterologous modules for efficient and versatile PCR-based gene targeting in Schizosaccharomyces pombe. Yeast. 1998; 14:943–951. 9717240 10.1002/(SICI)1097-0061(199807)14:10<943::AID-YEA292>3.0.CO;2-Y

[33] Bilokapic S., Strauss M., Halic M. Histone octamer rearranges to adapt to DNA unwrapping. Nat. Struct. Mol. Biol. 2018; 25:101–108. 29323273 10.1038/s41594-017-0005-5 PMC5800490

[34] Simon M.D., Chu F., Racki L.R., de la Cruz C.C., Burlingame A.L., Panning B., Narlikar G.J., Shokat K.M. The site-specific installation of methyl-lysine analogs into recombinant histones. Cell. 2007; 128:1003–1012. 17350582 10.1016/j.cell.2006.12.041 PMC2932701

[35] Wüthrich K. NMR studies of structure and function of biological macromolecules (Nobel Lecture). J. Biomol. NMR. 2003; 27:13–39. 15143746 10.1023/a:1024733922459

[36] Williamson M.P. Using chemical shift perturbation to characterise ligand binding. Prog. Nucl. Magn. Reson. Spectrosc. 2013; 73:1–16. 23962882 10.1016/j.pnmrs.2013.02.001

[37] Ekwall K., Olsson T., Turner B.M., Cranston G., Allshire R.C. Transient inhibition of histone deacetylation alters the structural and functional imprint at fission yeast centromeres. Cell. 1997; 91:1021–1032. 9428524 10.1016/s0092-8674(00)80492-4

[38] Brönner C., Salvi L., Zocco M., Ugolini I., Halic M. Accumulation of RNA on chromatin disrupts heterochromatic silencing. Genome Res. 2017; 27:1174–1183. 28404620 10.1101/gr.216986.116 PMC5495069

[39] Ishida M., Shimojo H., Hayashi A., Kawaguchi R., Ohtani Y., Uegaki K., Nishimura Y., Nakayama J.-I. Intrinsic nucleic acid-binding activity of Chp1 chromodomain is required for heterochromatic gene silencing. Mol. Cell. 2012; 47:228–241. 22727667 10.1016/j.molcel.2012.05.017

[40] Akoury E., Mukrasch M.D., Biernat J., Tepper K., Ozenne V., Mandelkow E., Blackledge M., Zweckstetter M. Remodeling of the conformational ensemble of the repeat domain of tau by an aggregation enhancer. Protein Sci. 2016; 25:1010–1020. 26940799 10.1002/pro.2911 PMC4838641

[41] Sgourakis N.G., Lange O.F., DiMaio F., André I., Fitzkee N.C., Rossi P., Montelione G.T., Bax A., Baker D. Determination of the structures of symmetric protein oligomers from NMR chemical shifts and residual dipolar couplings. J. Am. Chem. Soc. 2011; 133:6288–6298. 21466200 10.1021/ja111318m PMC3080108

[42] Shen Y., Bax A. Identification of helix capping and b-turn motifs from NMR chemical shifts. J. Biomol. NMR. 2012; 52:211–232. 22314702 10.1007/s10858-012-9602-0 PMC3357447

[43] Wang X., Paucek R.D., Gooding A.R., Brown Z.Z., Ge E.J., Muir T.W., Cech T.R. Molecular analysis of PRC2 recruitment to DNA in chromatin and its inhibition by RNA. Nat. Struct. Mol. Biol. 2017; 24:1028–1038. 29058709 10.1038/nsmb.3487 PMC5771497

[44] Kaneko S., Son J., Bonasio R., Shen S.S., Reinberg D. Nascent RNA interaction keeps PRC2 activity poised and in check. Genes Dev. 2014; 28:1983–1988. 25170018 10.1101/gad.247940.114 PMC4173153

[45] Rao V.K., Pal A., Taneja R. A drive in SUVs: From development to disease. Epigenetics. 2017; 12:177–186. 28106510 10.1080/15592294.2017.1281502 PMC5406210

[46] Casciello F., Windloch K., Gannon F., Lee J.S. Functional role of G9a histone methyltransferase in cancer. Front. Immunol. 2015; 6:487–499. 26441991 10.3389/fimmu.2015.00487 PMC4585248

