# Supplementary material for: Disordered region of H3K9 methyltransferase Clr4 binds the nucleosome and contributes to its activity
Source: Nucleic Acids Res. 2019 Jun 5;47(13):6726–36. doi: 10.1093/nar/gkz480 (PMC6649693; doi:10.1093/nar/gkz480)
Supplement: gkz480_Supplemental_File [file gkz480_supplemental_file.pdf]

**Supplementary Information**

# Disordered region of H3K9 methyltransferase Clr4 binds the nucleosome and contributes to its activity

Elias Akoury[1,2,3], Guoli Ma[1,7], Segolene Demolin[1,7], Cornelia Brönner[1,7], Manuel Zocco[1,4], Alexandre Cirilo[1], Nives Ivic[1,5], Mario Halic[1,6]

[1] Department of Biochemistry, Gene Center, Ludwig-Maximilians-Universität LMU, Feodor-Lynen-Strasse 25, 81377 Munich, Germany

[2] Department of Chemistry, Faculty of Chemistry and Pharmacy, Ludwig-Maximilians-Universität LMU, Butenandtstrasse 5-13, 81377 Munich, Germany

[3] Department of Natural Sciences, Lebanese American University, Beirut 1102-2801, Lebanon

[4] Université Libre de Bruxelles, IRIBHM, Brussels B-1070, Belgium

[5] Department of Physical Chemistry, Rudjer Boskovic Institute, Zagreb, Croatia

[6] Department of Structural Biology, St. Jude Children's Research Hospital, 263 Danny Thomas Place, Memphis, TN, 38105, USA

[7] These authors contributed equally

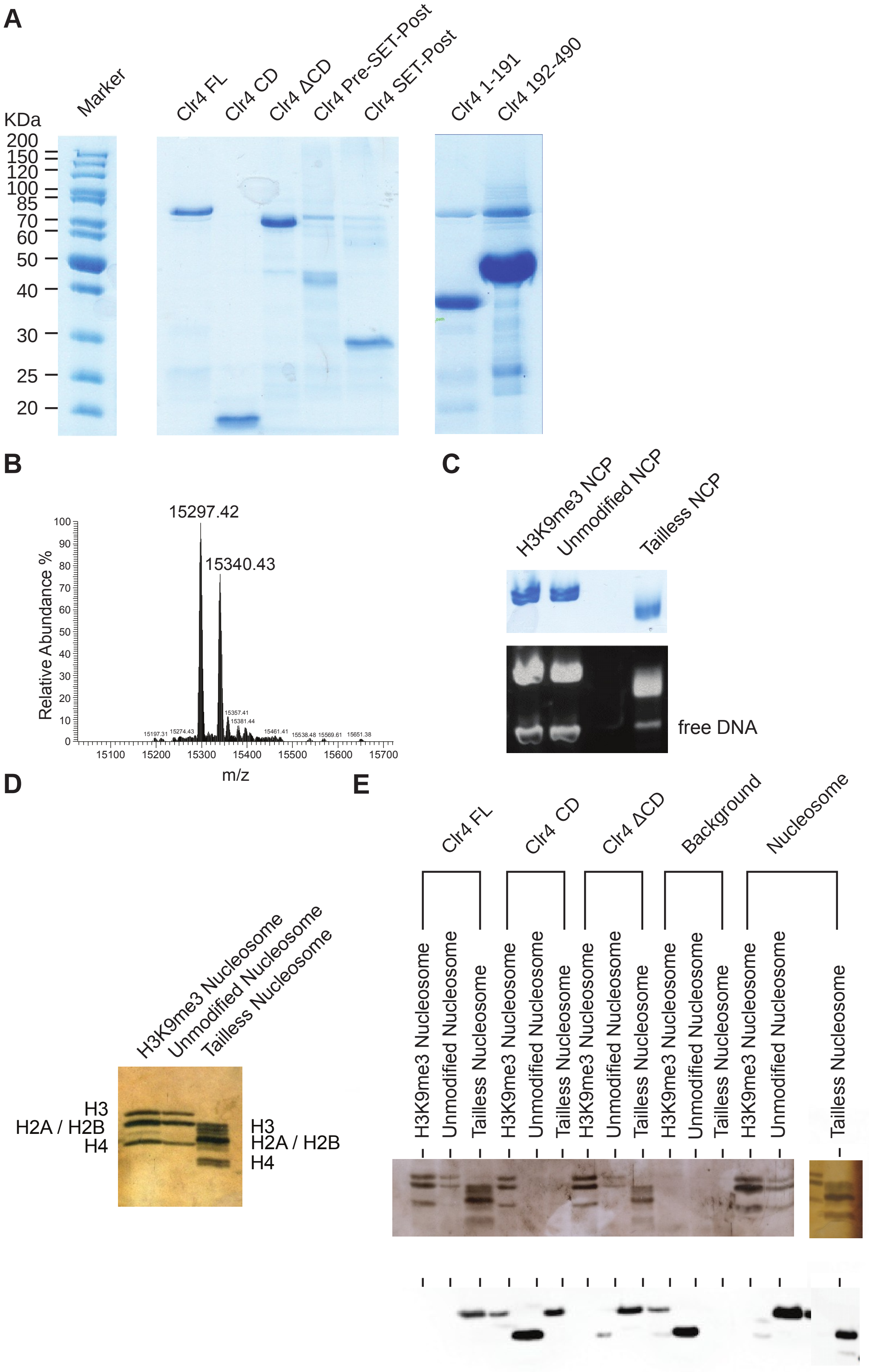
A
Marker
Clr4 FL
Clr4 CD
Clr4 ΔCD
Clr4 Pre-SET-Post
Clr4 SET-Post
Clr4 1-191
Clr4 192-490
KDa
200
150
120
100
85
70
60
50
40
30
25
20
B
15297.42
15340.43
Relative Abundance %
m/z
C
H3K9me3 NCP
Unmodified NCP
Tailless NCP
free DNA
D
H3K9me3 Nucleosome
Unmodified Nucleosome
Tailless Nucleosome
H3
H2A / H2B
H4
E
Clr4 FL
Clr4 CD
Clr4 ΔCD
Background
Nucleosome
H3K9me3 Nucleosome
Unmodified Nucleosome
Tailless Nucleosome

**Figure S1**

**Figure S1. Binding assays of Clr4 constructs with nucleosome.**

**(A)** Coomassie-stained SDS-PAGE of purified FLAG-tagged protein constructs: Clr4 FL, Clr4 CD, ΔCD, Clr4 Pre-SET-Post, Clr4 SET-post and the two constructs Clr4_1-191 and Clr4_192-490.

**(B)** Mass Spectrometry (MS) analysis of H3KC9me3 histone showing a peak at 15297 Da. The MS analysis is consistent with previously published results.

**(C)** Native gel of methylated, unmodified and tailless nucleosomes. The agarose gel shows nucleosome-bound DNA and free DNA.

**(D)** Silver stained 15% SDS-PAGE showing assembly of methylated, unmodified and tailless nucleosomes.

**(E)** Binding of FLAG-tagged Clr4 FL, Clr4 CD, Clr4 ΔCD constructs to all three types of nucleosomes as shown by silver stained SDS-PAGE and western blot analysis. Anti-FLAG antibodies designate the light and heavy chains from the resin.

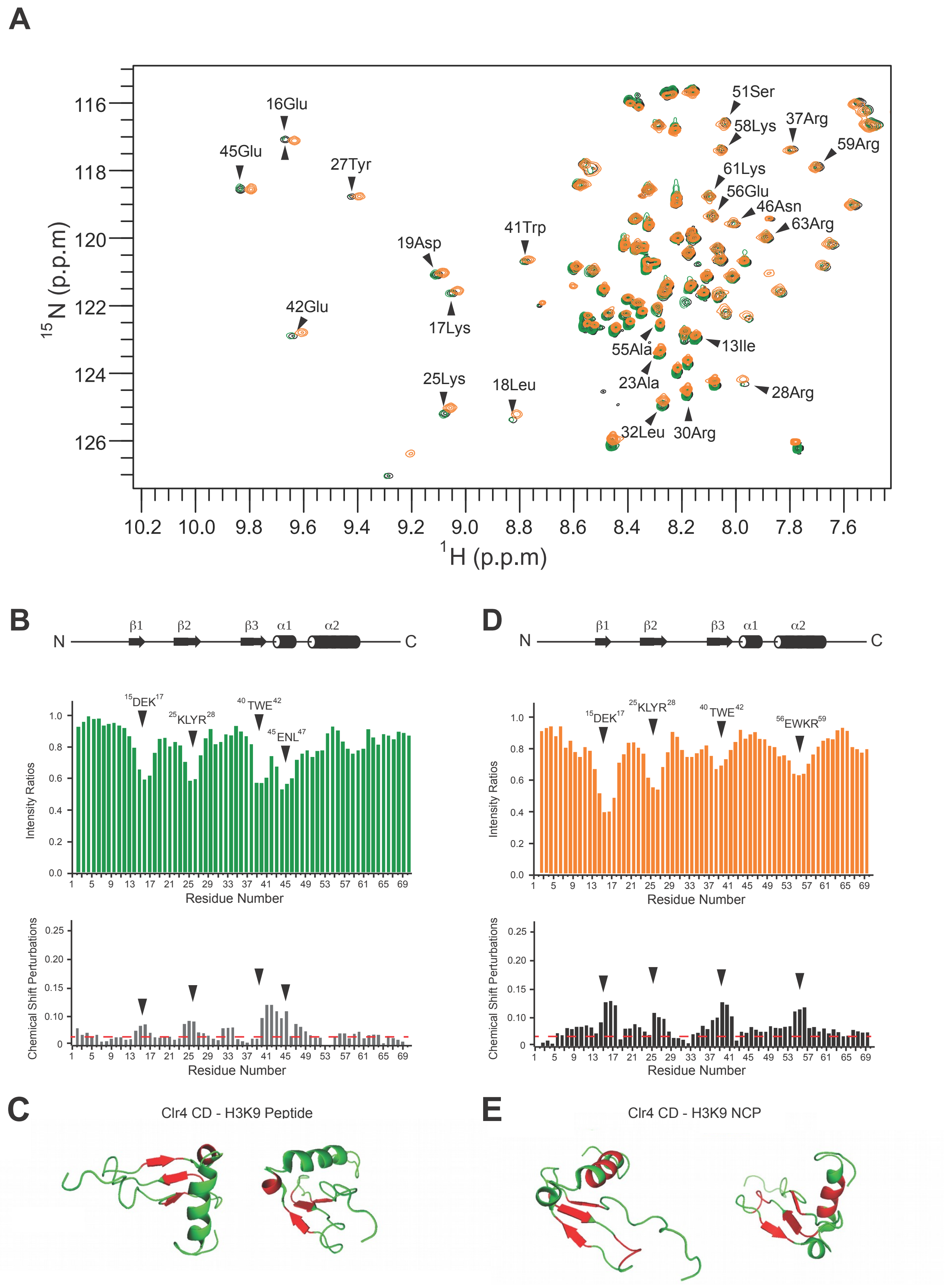

A
116
118
120
122
124
126
$^{15}$N (p.p.m)
10.2 10.0 9.8 9.6 9.4 9.2 9.0 8.8 8.6 8.4 8.2 8.0 7.8 7.6
$^{1}$H (p.p.m)
16Glu
45Glu
27Tyr
19Asp
41Trp
17Lys
42Glu
25Lys
18Leu
51Ser
58Lys
37Arg
59Arg
61Lys
56Glu
46Asn
63Arg
13Ile
55Ala
23Ala
28Arg
32Leu
30Arg
B
N
β1 β2 β3 α1 α2
C
$^{15}$DEK$^{17}$
$^{25}$KLYR$^{28}$
$^{40}$TWE$^{42}$
$^{45}$ENL$^{47}$
Intensity Ratios
Residue Number
Chemical Shift Perturbations
Residue Number
D
N
β1 β2 β3 α1 α2
C
$^{15}$DEK$^{17}$
$^{25}$KLYR$^{28}$
$^{40}$TWE$^{42}$
$^{56}$EWKR$^{59}$
Intensity Ratios
Residue Number
Chemical Shift Perturbations
Residue Number
C
Clr4 CD - H3K9 Peptide
E
Clr4 CD - H3K9 NCP


**Figure S2**

**Figure S2. The Interaction between Clr4-Chromodomain and the H3KC9me3 nucleosome.**

**(A)** Two-dimensional $^{1}H$-$^{15}N$ HSQC spectra of isotope-labeled Clr4 CD in the absence (black) and presence of unlabeled H3KC9me3 nucleosome (orange) and H3K9 methylated peptide (green). The insets highlight perturbation and broadening in the chemical shifts of certain CD resonances.

**(B)** Intensity ratio plots and chemical shift perturbations of Clr4 CD in the presence of the H3K9 methylated peptide. Intensity ratios and weighted chemical shift perturbations correspond to the backbone amide resonances. The residue patches $^{15}DEK^{17}$, $^{25}KLYR^{28}$ and $^{40}TWE^{42}$ showed significant perturbations and decrease in signal intensities in presence of the H3K9 methylated peptide.

**(C)** The residues involved in the nucleosome binding are highlighted on the Clr4 CD PDB structure (1G6Z).

**(D)** Intensity ratio plots and chemical shift perturbations of Clr4 CD in the presence of the H3KC9me3 nucleosome. Upon binding of the H3KC9me3 nucleosome, the residues $^{15}DEK^{17}$, $^{25}KLYR^{28}$, $^{40}TWE^{42}$ and $^{56}EWKR^{59}$ showed significant perturbations and decrease in signal intensities.

**(E)** The residues involved in the nucleosome binding are highlighted on the Clr4 CD PDB structure (1G6Z).

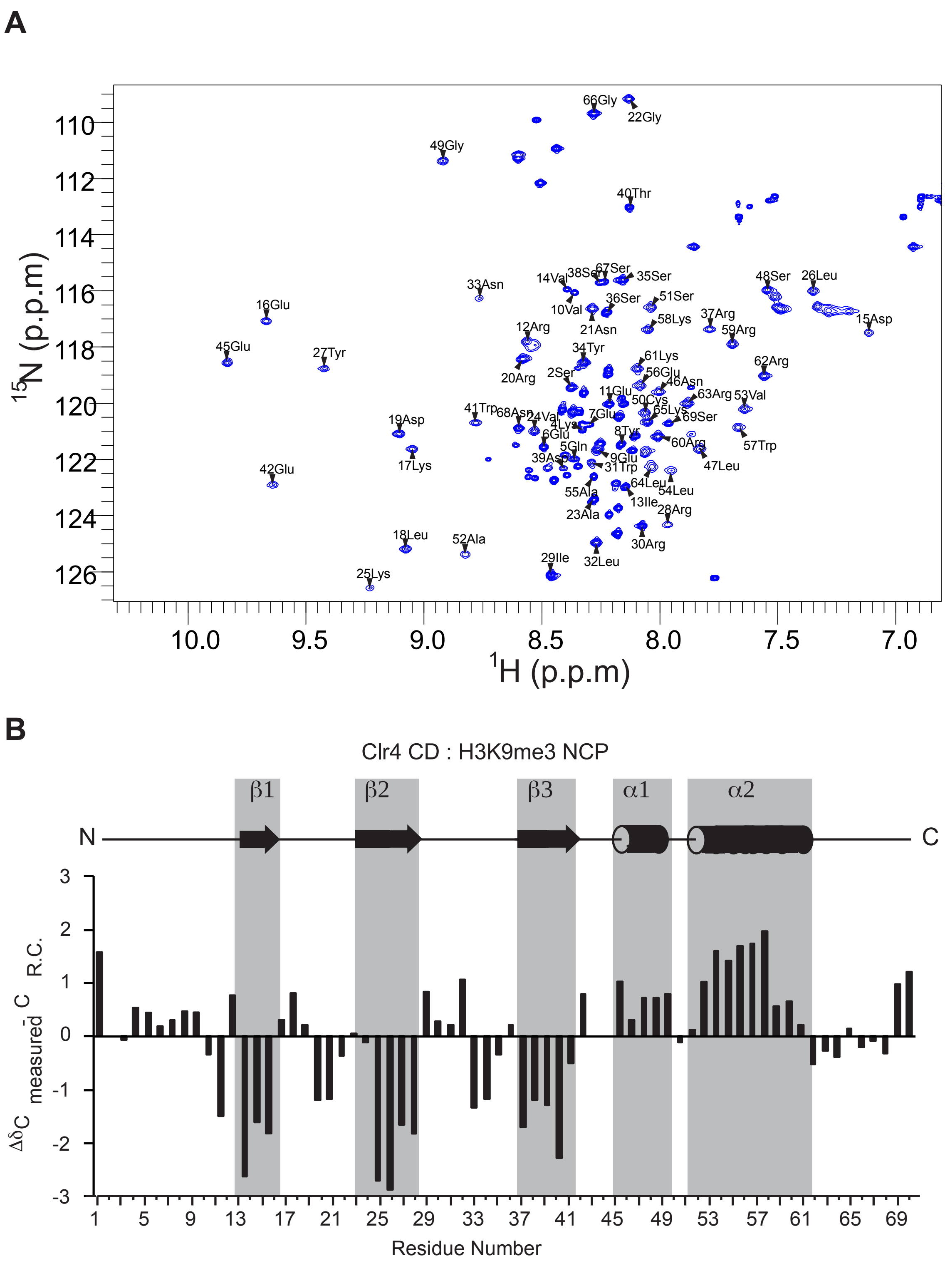
A
$^{15}$N (p.p.m)
$^{1}$H (p.p.m)
B
Clr4 CD : H3K9me3 NCP
β1
β2
β3
α1
α2
N
C
$\Delta\delta C_{measured} - C_{R.C.}$
Residue Number

**Figure S3**

**Figure S3. The Interaction between Clr4 CD and the H3KC9me3 nucleosome**

**(A)** 2D HSQC Spectrum of Clr4 CD with the NMR chemical shift assignment of the amide backbone.

**(B)** Secondary structure elements of Clr4 CD-H3CK9me3 nucleosome complex. The indicated secondary structure is based on the Cα NMR chemical shifts analysis and represents differences between measured Cα and random coil (RC) chemical shifts as a function of the primary sequence in the Clr4 CD. The secondary structure elements are highlighted in grey. Cα atoms in α-helices and β-sheets have positive and negative secondary chemical shifts, respectively.

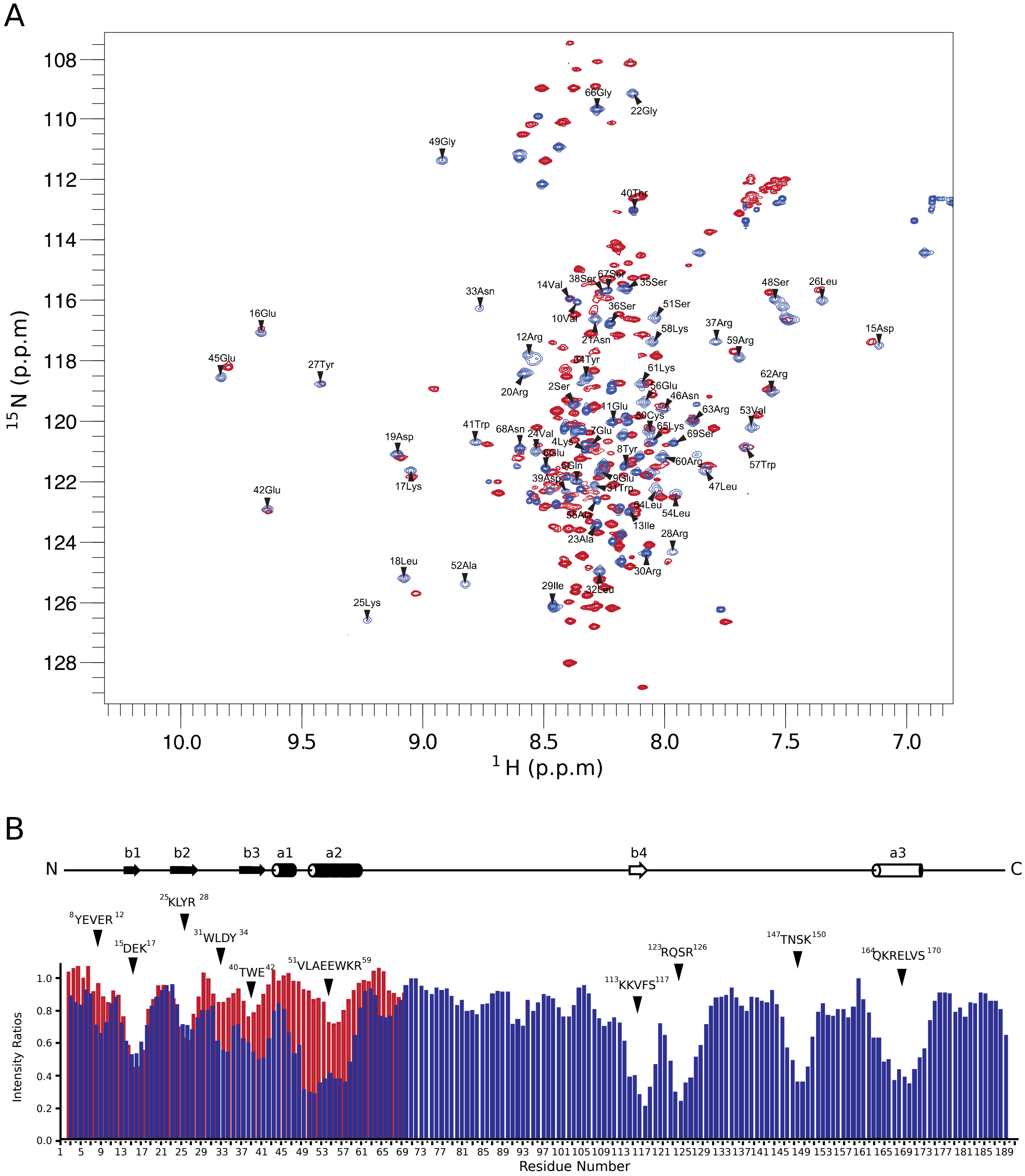

A
15N (p.p.m)
1H (p.p.m)
108
110
112
114
116
118
120
122
124
126
128
10.0
9.5
9.0
8.5
8.0
7.5
7.0
66Gly
22Gly
49Gly
40Thr
33Asn
16Glu
45Glu
27Tyr
14Val
38Ser
67Ser
35Ser
48Ser
26Leu
15Asp
10Val
36Ser
51Ser
58Lys
37Arg
59Arg
21Asn
12Arg
44Tyr
61Lys
62Arg
20Arg
2Ser
56Glu
46Asn
11Glu
63Arg
53Val
50Cys
65Lys
69Ser
41Trp
68Asn
24Val
7Glu
4Lys
19Asp
8Tyr
57Trp
60Arg
47Leu
17Lys
39Asp
9Glu
31Trp
42Glu
34Leu
54Leu
13Ile
55Ala
23Ala
28Arg
30Arg
18Leu
52Ala
29Ile
32Leu
25Lys
B
N
C
b1
b2
b3
a1
a2
b4
a3
8YEVER12
25KLYR28
15DEK17
31WLDY34
40TWE42
51VLAEEWKR59
113KKVFS117
123RQSR126
147TNSK150
164QKRELVS170
Intensity Ratios
Residue Number

**Figure S4. Comparison of Clr4 CD and Clr4 1-191 binding to H3KC9me3 nucleosome.**

**(A)** Overlay of 2D $^{1}H$-$^{15}N$ HSQC spectra of Clr4 CD (blue) and Clr4_1-191 (red) showing minor changes in chemical shift perturbations between both constructs. These data show that the global fold of the chromodomain is retained independent of the additional disordered regions between 70 and 191.

**(B)** Intensity ratio plots of Clr4 CD (red) and Clr4_1-191 (blue) upon interaction with H3KC9me3 nucleosome. The residue patches $^{15}$DEK$^{17}$ and $^{25}$KLYR$^{28}$ show similar decrease in signal intensities for Clr4 CD and Clr4_1-191. Residues $^{40}$TWE$^{42}$ and $^{51}$VLAEEWKR$^{59}$ show stronger decrease in signal intensities for Clr4_1-191 than for Clr4 CD.

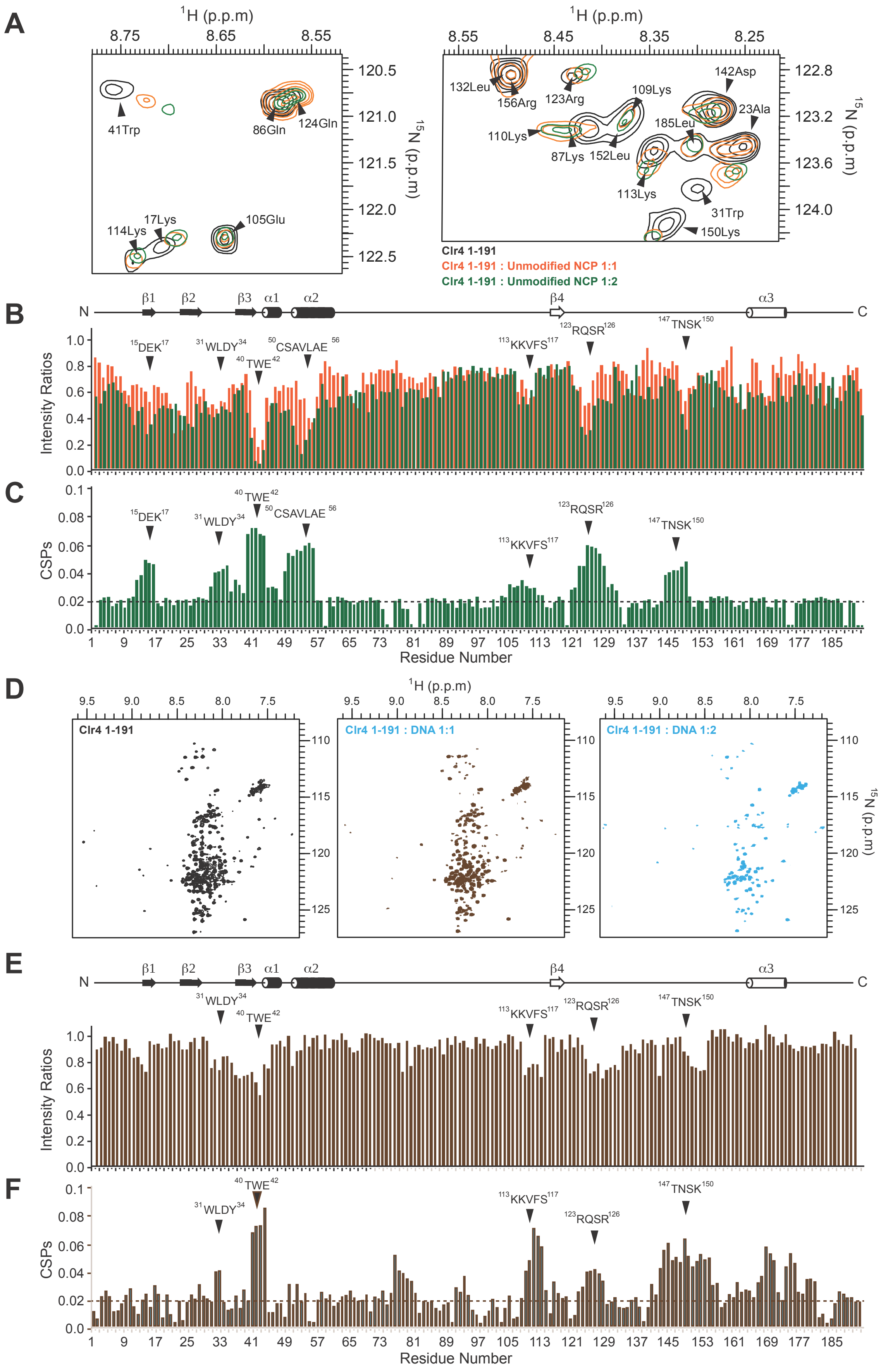

A
1H (p.p.m)
15N (p.p.m)
41Trp
86Gln
124Gln
17Lys
114Lys
105Glu
132Leu
156Arg
123Arg
109Lys
142Asp
23Ala
185Leu
110Lys
87Lys
152Leu
113Lys
31Trp
150Lys
Clr4 1-191
Clr4 1-191 : Unmodified NCP 1:1
Clr4 1-191 : Unmodified NCP 1:2
B
Intensity Ratios
15DEK17
31WLDY34
40TWE42
50CSAVLAE56
113KKVFS117
123RQSR126
147TNSK150
C
CSPs
Residue Number
D
Clr4 1-191
Clr4 1-191 : DNA 1:1
Clr4 1-191 : DNA 1:2
E
Intensity Ratios
F
CSPs
Residue Number


**Figure S5**

**Figure S5. The Interaction of Clr4 1-191 with the unmodified nucleosome and DNA.**

**(A)** Insets from two-dimensional $^{1}$H-$^{15}$N HSQC spectra of isotope-labeled Clr4_1-191 construct in the absence (black) and presence of increased ratios of unlabeled unmodified nucleosome; Clr4_1-191:nucleosome 1:1 (orange) and Clr4_1-191:nucleosome 1:2 (green). Several residues show perturbation in their chemical shifts and are highlighted in the

**(B)** Intensity ratio plots and **(C)** Chemical shift perturbations upon Clr4_1-191 binding to unmodified nucleosomes. Intensity ratios and weighted chemical shift perturbations correspond to the backbone amide resonances. The residue patches of the chromodomain ($^{15}$DEK$^{17}$, $^{25}$KLYR$^{28}$, $^{40}$TWE$^{42}$, $^{51}$VLAEEW$^{57}$) and the residue patches in the unfolded region ($^{113}$KKVFS$^{117}$, $^{123}$RQSR$^{126}$, $^{147}$TNSK$^{150}$) showed significant perturbations and decrease in signal intensities in presence of the unmodified nucleosome.

**(D)** Two-dimensional $^{1}$H-$^{15}$N HSQC spectra of isotope-labeled Clr4_1-191 construct were measured in the absence (black) and presence of DNA; Clr4_1-191:DNA 1:1 (brown) and Clr4_1-191:DNA 1:2 (light blue). Similarly, several residues show perturbation in their chemical shifts and are highlighted in the **(E)** intensity ratio plots and **(F)** chemical shift perturbations. The residues $^{31}$WLDY$^{34}$ , $^{40}$TWE$^{42}$, $^{113}$KKVFS$^{117}$, $^{123}$RQSR$^{126}$, and $^{147}$TNSK$^{150}$ showed significant perturbations and decrease in signal intensities upon DNA binding.

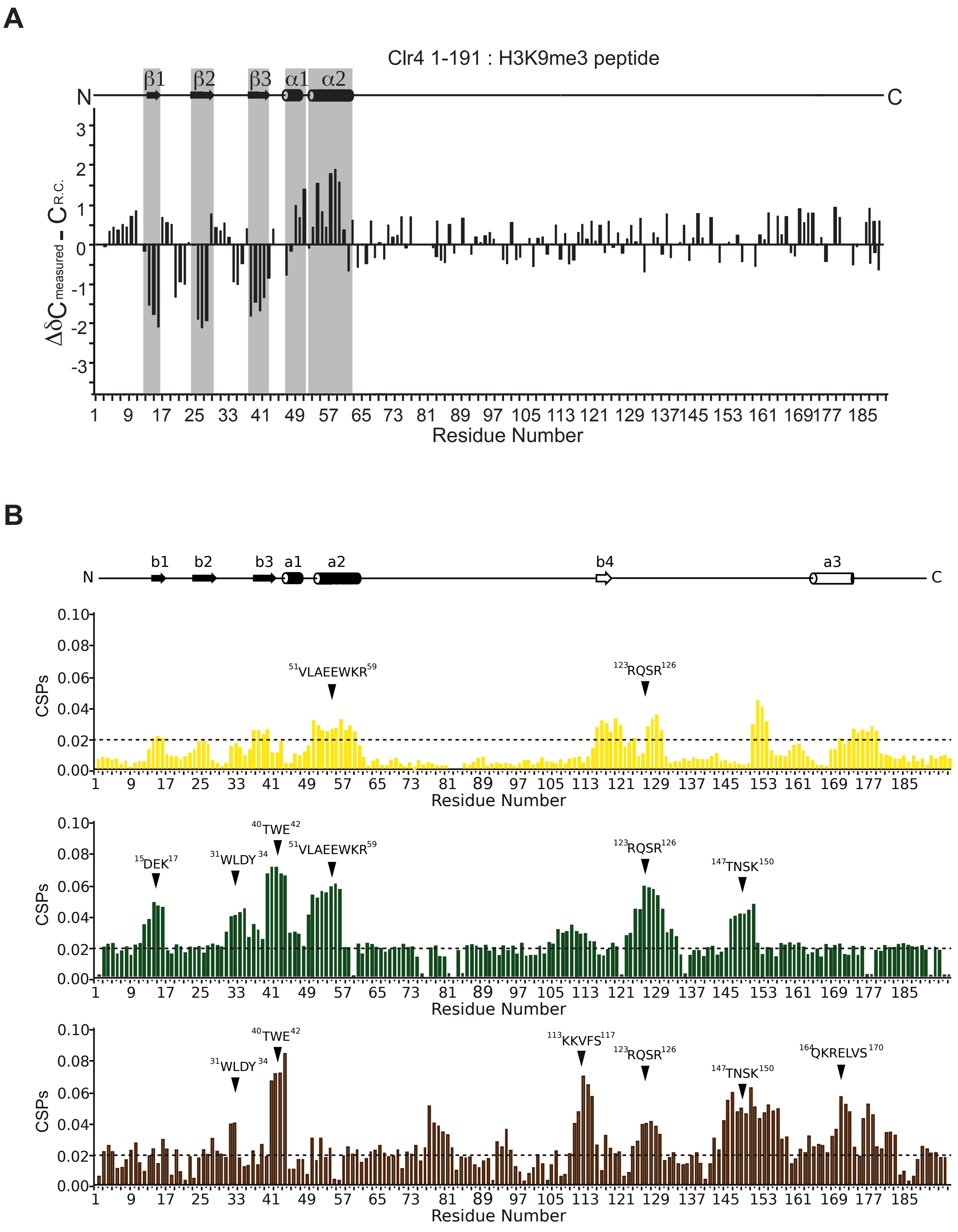
A
Clr4 1-191 : H3K9me3 peptide
β1 β2 β3 α1 α2
N
C
ΔδC measured - C R.C.
Residue Number
B
b1 b2 b3 a1 a2 b4 a3
CSPs
51VLAEEWKR59
123RQSR126
15DEK17
31WLDY34
40TWE42
147TNSK150
113KKVFS117
164QKRELVS170
Residue Number

**Figure S6**

**Figure S6. The Interaction of Clr4 1-191 with the H3KC9me3 and unmodified nucleosome, and DNA.**

**(A)** Secondary structure elements of free Clr4 1-191. The indicated secondary structure is based on the Cα NMR chemical shifts analysis and represents differences between measured Cα and random coil (RC) chemical shifts as a function of the primary sequence in the Clr4. The secondary structure elements are highlighted in grey.

**(B)** Weighted Chemical Shift Perturbations (CSPs) upon Clr4_1-191 binding to H3KC9me3 (yellow), unmodified (green) and DNA (brown) as a function of residue number. Residue patches along the chromodomain and the unfolded region represent the binding sites of the protein on the three different species.

A

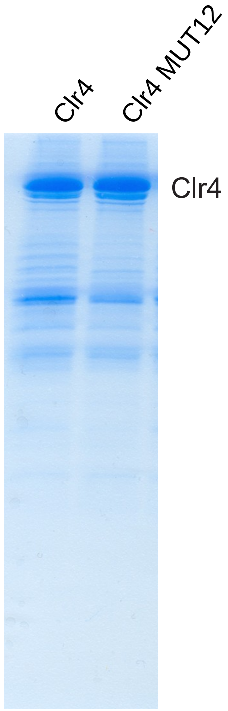


## Figure S7

**Figure S7. Interaction of Clr4 disordered region with the nucleosome promotes H3K9 methylation and heterochromatin establishment.**

**(A)** Coomassie-stained SDS-PAGE of purified FLAG-tagged wild type and MUT12 Clr4.

**Table S1. List of plasmids used in this study**

| | |
|---|---|
| p406 | pET3- Xenopus laevis histone H2A |
| p407 | pET3- Xenopus laevis histone H2B |
| p408 | pET3- Xenopus laevis histone H3 |
| p409 | pET3- Xenopus laevis histone H4 |
| p496 | pET30a- 6His-3FLAG Clr4 Full-length |
| p657 | pET30a- 6His-3FLAG Clr4 Chromodomain (residues 1-490) |
| p641 | pET30a- 6His-3FLAG Clr4 Delta Chromodomain (residues 70-490) |
| p645 | pET30a- 6His-3FLAG Clr4 PRE-SET-POST (residues 258-490) |
| p657 | pET30a- 6His-3FLAG Clr4 SET-POST (residues 328-490) |
| p715 | pET30a- 6His-3FLAG Clr4 1-191 (residues 1-191) |
| p717 | pET30a- 6His-3FLAG Clr4 192-490 (residues 192-490) |
| p1167 | pET30a- 6His-3FLAG Clr4 Mut12 (residues147-150 & 164-170) |
| p420 | 8X 145bp 601 DNA sequence |

**Table S2. List of oligonucleotides used in this study**

| Oligonucleotide | Sequence |
|---|---|
| 470F Clr4 CD | GCAGATTATAAAGATGACGAC |
| 470R Clr4 CD | GTCGGAGTTACTTCCCT |
| 448F Clr4 Delta CD | GACTCGGATTCACCGC |
| 448R Clr4 Delta CD | GGATCCGATATCAGCCATG |
| 449F Clr4 258-590 | TCTGGATGCAATTGCTCG |
| 450F Clr4 328-490 | CTTCCGCTTGAAATATTTAAAAC |
| 451F Clr4 473-490 | CTTCGCCGGCAGTGC |
| 543F Clr4 1-191 | GCAGATTATAAAGATGACGACG |
| 543R Clr4 1-191 | AGGGTTTCGCGGTTTTG |
| 556F Clr4 192-490 | TCCAAACTTGACTCTTATACC |
| 556R Clr4 192-490 | CATGGATCCGATATCAGC |
| 893F Clr4 Mut12 164-170 | GGAGGAAATTCTATTAAAGAAGCTAC |
| 893R Clr4 Mut12 164-170 | ACCGCTAGATTCCTCCTTCACTTC |
| 894F Clr4 Mut12 147-150 | TCGGGAAAGCTGGGGAAGACGAG |
| 894R Clr4 Mut12 147-150 | GCTATGAAGACTATCGTCTAATCAAAC |

**Table S3. Chemical Shift Assignments of Clr4 1-191 Construct**

| **Residue** | **$d_{HN}$** | **$d_{CA}$** | **$d_{N}$** |
|---|---|---|---|
| 4 Lys | 8.33 | 56.57 | 120.78 |
| 5 Gln | 8.37 | 56.33 | 121.91 |
| 7 Glu | 8.32 | 54.84 | 120.95 |
| 8 Tyr | 8.18 | 62.61 | 121.39 |
| 9 Glu | 8.26 | 53.14 | 121.32 |
| 10 Val | 8.36 | 62.07 | 115.79 |
| 12 Arg | 8.52 | 58.53 | 117.83 |
| 13 Ile | 8.16 | 61.56 | 123.04 |
| 22 Gly | 8.12 | 44.10 | 108.75 |
| 23 Ala | 8.28 | 55.31 | 123.48 |
| 30 Arg | 8.08 | 55.93 | 124.03 |
| 31 Trp | 8.29 | 57.08 | 121.91 |
| 34 Tyr | 8.33 | 56.64 | 118.51 |
| 36 Ser | 8.23 | 56.34 | 116.77 |
| 42 Glu | 9.63 | 55.82 | 122.45 |
| 49 Gly | 8.95 | 43.59 | 111.22 |
| 54 Leu | 7.99 | 61.31 | 122.51 |
| 62 Arg | 7.56 | 64.14 | 119.10 |
| 64 Leu | 8.03 | 55.64 | 122.36 |
| 70 Asp | 8.44 | 54.62 | 122.53 |
| 71 Ser | 8.14 | 56.60 | 116.88 |
| 72 Asp | 8.50 | 56.06 | 121.93 |
| 77 Ala | 8.49 | 50.77 | 124.70 |
| 85 Arg | 8.42 | 56.57 | 122.93 |
| 86 Gln | 8.34 | 54.72 | 120.25 |
| 87 Lys | 8.48 | 56.36 | 123.50 |
| 92 Thr | 8.12 | 61.85 | 115.71 |
| 93 Ser | 8.41 | 59.81 | 116.25 |
| 94 Lys | 8.42 | 56.60 | 125.18 |
| 96 Val | 8.19 | 62.30 | 116.86 |
| 98 Arg | 8.46 | 56.59 | 121.77 |
| 99 Ser | 8.30 | 58.79 | 115.44 |
| 101 Arg | 8.53 | 56.09 | 121.75 |
| 102 Phe | 8.29 | 58.82 | 116.16 |
| 104 Arg | 8.40 | 56.54 | 122.93 |
| 105 Glu | 8.52 | 56.58 | 121.77 |
| 106 Leu | 8.26 | 56.82 | 122.12 |
| 108 Val | 8.28 | 62.08 | 116.27 |
| 109 Lys | 8.36 | 56.59 | 122.93 |

| | | | |
|---|---|---|---|
| 110 Lys | 8.45 | 58.33 | 123.57 |
| 111 Glu | 8.26 | 56.85 | 121.58 |
| 113 Lys | 8.36 | 56.60 | 122.93 |
| 115 Val | 8.96 | 64.63 | 119.15 |
| 116 Phe | 8.42 | 56.63 | 125.75 |
| 117 Ser | 8.04 | 54.48 | 117.92 |
| 118 Ser | 8.46 | 58.58 | 117.78 |
| 119 Gln | 8.42 | 54.34 | 120.35 |
| 120 Thr | 8.21 | 61.84 | 115.92 |
| 121 Thr | 8.24 | 62.33 | 115.00 |
| 122 Lys | 8.33 | 55.26 | 125.75 |
| 123 Arg | 8.46 | 56.61 | 122.47 |
| 124 Gln | 8.26 | 56.36 | 120.84 |
| 126 Arg | 8.42 | 56.57 | 122.93 |
| 127 Lys | 8.42 | 56.60 | 122.29 |
| 130 Thr | 8.19 | 60.09 | 113.60 |
| 131 Ala | 8.33 | 52.62 | 126.45 |
| 132 Leu | 8.53 | 57.94 | 121.97 |
| 133 Thr | 8.17 | 61.82 | 113.25 |
| 134 Thr | 8.20 | 61.87 | 115.00 |
| 135 Asn | 8.35 | 56.56 | 119.73 |
| 136 Asp | 8.31 | 54.32 | 125.34 |
| 137 Thr | 8.42 | 55.30 | 109.57 |
| 138 Ser | 8.33 | 59.01 | 116.39 |
| 139 Ile | 8.10 | 62.09 | 121.61 |
| 140 Ile | 8.28 | 61.39 | 126.29 |
| 141 Leu | 8.38 | 56.67 | 124.06 |
| 142 Asp | 8.31 | 55.39 | 123.54 |
| 143 Asp | 8.15 | 53.17 | 123.00 |
| 145 Leu | 8.36 | 56.56 | 125.75 |
| 148 Asn | 8.45 | 58.55 | 119.49 |
| 149 Ser | 8.45 | 58.57 | 118.59 |
| 150 Lys | 8.41 | 56.58 | 125.18 |
| 151 Lys | 8.45 | 58.34 | 123.50 |
| 153 Gly | 8.41 | 45.32 | 109.79 |
| 154 Lys | 8.34 | 56.35 | 122.34 |
| 155 Thr | 8.23 | 61.62 | 114.64 |
| 156 Arg | 8.52 | 56.60 | 122.93 |
| 157 Asn | 8.33 | 52.81 | 119.27 |
| 158 Glu | 8.75 | 51.02 | 122.52 |
| 158 Glu | 8.46 | 56.82 | 121.94 |
| 160 Lys | 8.54 | 61.85 | 122.46 |

| | | | |
|---|---|---|---|
| 161 Glu | 8.30 | 55.32 | 121.41 |
| 162 Glu | 8.38 | 54.57 | 121.22 |
| 165 Lys | 7.80 | 58.09 | 120.24 |
| 167 Glu | 8.57 | 56.43 | 121.85 |
| 169 Val | 8.26 | 64.32 | 121.09 |
| 171 Asn | 8.44 | 58.54 | 119.48 |
| 172 Ser | 8.43 | 59.07 | 118.66 |
| 173 Ile | 8.17 | 61.58 | 122.35 |
| 174 Lys | 8.33 | 55.16 | 125.75 |
| 176 Ala | 8.45 | 55.00 | 127.66 |
| 176 Ala | 8.42 | 52.78 | 125.33 |
| 178 Ser | 8.38 | 60.37 | 116.30 |
| 181 Thr | 8.23 | 62.22 | 114.69 |
| 183 Ser | 8.41 | 59.16 | 116.79 |
| 184 Ile | 8.17 | 61.52 | 122.35 |
| 185 Leu | 8.40 | 56.56 | 123.50 |
| 186 Thr | 8.17 | 61.82 | 113.47 |
| 187 Lys | 8.27 | 60.87 | 126.41 |
| 190 Asn | 8.22 | 55.64 | 119.40 |
